# Surveillance of *pfhrp2* and *pfhrp3* gene deletions among symptomatic *Plasmodium falciparum* malaria patients in Central Vietnam

**DOI:** 10.1186/s12936-022-04399-w

**Published:** 2022-12-05

**Authors:** Ngo Duc Thang, Eduard Rovira-Vallbona, Nguyen Thi Huong Binh, Dang Viet Dung, Nguyen Thi Hong Ngoc, Tran Khanh Long, Tran Thanh Duong, Nicholas J. Martin, Kimberly A. Edgel

**Affiliations:** 1 National Institute of Malariology, Parasitology and Entomology, Hanoi, Vietnam; 2 Vysnova Partners, Landover, MD USA; 3 U.S. Naval Medical Research Unit TWO, Singapore, Singapore

**Keywords:** Malaria, *Plasmodium falciparum*, Diagnostics, Rapid Diagnostic Test, Histidine rich protein 2, Histidine rich protein 3, Gene deletion, Surveillance, Vietnam

## Abstract

**Background:**

Malaria rapid diagnostic tests (RDTs) remain the main point-of-care tests for diagnosis of symptomatic *Plasmodium falciparum* malaria in endemic areas. However, parasites with gene deletions in the most common RDT target, histidine rich protein 2 (*pfhrp2*/HRP2), can produce false-negative RDT results leading to inadequate case management. The objective of this study was to determine the prevalence of *hrp2/3* deletions causing false-negative RDT results in Vietnam (Gia Lai and Dak Lak provinces).

**Methods:**

Individuals presenting with malaria symptoms at health facilities were screened for *P. falciparum* infection using light microscopy and HRP2-RDT (SD Bioline Malaria Antigen Pf/Pv RDT, Abbott). Microscopically confirmed *P. falciparum* infections were analysed for parasite species by 18S rRNA qPCR, and *pfhrp2* and *pfhrp3* exon2 deletions were investigated by nested PCR. *pfhrp2* amplicons were sequenced by the Sanger method and HRP2 plasma levels were determined by enzyme-linked immunosorbent assay (ELISA).

**Results:**

The prevalence of false-negative RDT results among symptomatic cases was 5.6% (15/270). No *pfhrp2* and *pfhrp3* deletions were identified. False-negative RDT results were associated with lower parasite density (p = 0.005) and lower HRP2 plasma concentrations (p < 0.001), as compared to positive RDT.

**Conclusions:**

The absence of *hrp2/3* deletions detected in this survey suggests that HRP2-based malaria RDTs remain effective for the diagnosis of symptomatic *P. falciparum* malaria in Central Vietnam.

**Supplementary Information:**

The online version contains supplementary material available at 10.1186/s12936-022-04399-w.

## Background

Malaria rapid diagnostic tests (RDT) play a key role in malaria case management, as they provide immediate diagnosis of malaria infections, especially in rural settings or in the absence of expert microscopy. RDT are lateral flow immunochromatographic tests designed to detect *Plasmodium* antigens present in human blood. The most commonly used RDT for *Plasmodium falciparum* targets histidine rich protein 2 (HRP2), an abundant secreted protein that is not present in other *Plasmodium* parasite species [1, 2]. These RDT are more sensitive and heat-stable than RDT detecting other antigens such as lactate-dehydrogenase (LDH) or aldolase. Importantly, antibodies in the test strip of HRP2-RDT may also cross-react with another antigen of the HRP family, namely HRP3, due to strong similarities in the amino acid repeat sequences [3].

Monitoring the accuracy of the RDT at point-of-care is critical for effective case management. Parasites with deletions in *pfhrp2* and/or *pfhrp3* genes can go undetected by HRP2-RDTs and are cause of concern in malaria endemic countries [4]. There are other common causes of false-negative RDT results, including product and storage quality, operator errors or parasite densities below the detection threshold of the device. However, some of these causes can be addressed with improved transport and storage of RDTs as well as end user training. The identification of *P. falciparum* parasites with *hrp2/3* gene deletions was first reported in the Peruvian Amazon in 2010, where *hrp2*-deleted parasites now constitute more than 50% of *P. falciparum* infections [5, 6]. Besides South America, the prevalence of *hrp2*/*3* deletions is highest in Eritrea and Djibouti (> 80%) [7, 8], prompting changes in national diagnostic guidelines towards LDH-based RDT. Data on *hrp2*/*3* deletions through systematic surveillance studies is limited for other countries in Africa and Asia [6].

Given the relatively quick emergence of such parasite populations and the importance of HRP2-based detection for malaria control, the World Health Organization (WHO) is urging countries to assess the prevalence of *hrp2/3* gene deletions causing false-negative tests. Prioritized areas include those with recognized discordance between HRP2-RDT and microscopy, with non-representative or sporadic reports of *hrp2/3* deletions in the country, or those that neighbour an area where frequent *hrp2/3* deletions have been identified [9]. If the prevalence of deletions known to cause false-negative RDT results among symptomatic individuals is > 5%, switching to non-HRP2-based RDT is recommended.

Vietnam has achieved a remarkable reduction in malaria cases in the past decade. *Plasmodium falciparum* and *Plasmodium vivax* remain endemic in Central and Southern provinces, with the former representing 60–70% of reported cases [10]. HRP2-RDT are the main diagnostic tool for *P. falciparum* at community health facilities, yet baseline information on prevalence of *hrp2/3* deletions is very limited. Global genomic surveillance conducted by MalariaGen Pf3K project (Wellcome Sanger Institute) reported a 4.6% prevalence of *pfhrp3*-deletions but no *pfhrp2*-deletions among Vietnam samples (n = 216) [11]. The present survey was designed to determine the prevalence of deletions in *hrp*2/3 genes causing false-negative RDT results among symptomatic *P. falciparum* cases in Gia Lai and Dak Lak, the two provinces with the highest malaria burden in Vietnam.

## Methods

### Study design

A cross-sectional multi-site study was conducted on individuals clinically suspected of having *P. falciparum* malaria seeking medical care at health facilities. The study was designed considering WHO guidelines for surveillance of *hrp2/3* deletions published in 2018 [9]. Screening for *P. falciparum* infection was conducted using routine HRP2-based RDT in accordance with National Malaria Control Programme malaria diagnosis policy, together with light microscopy (LM) as a confirmatory test.

### Study site

**Fig. 1:**
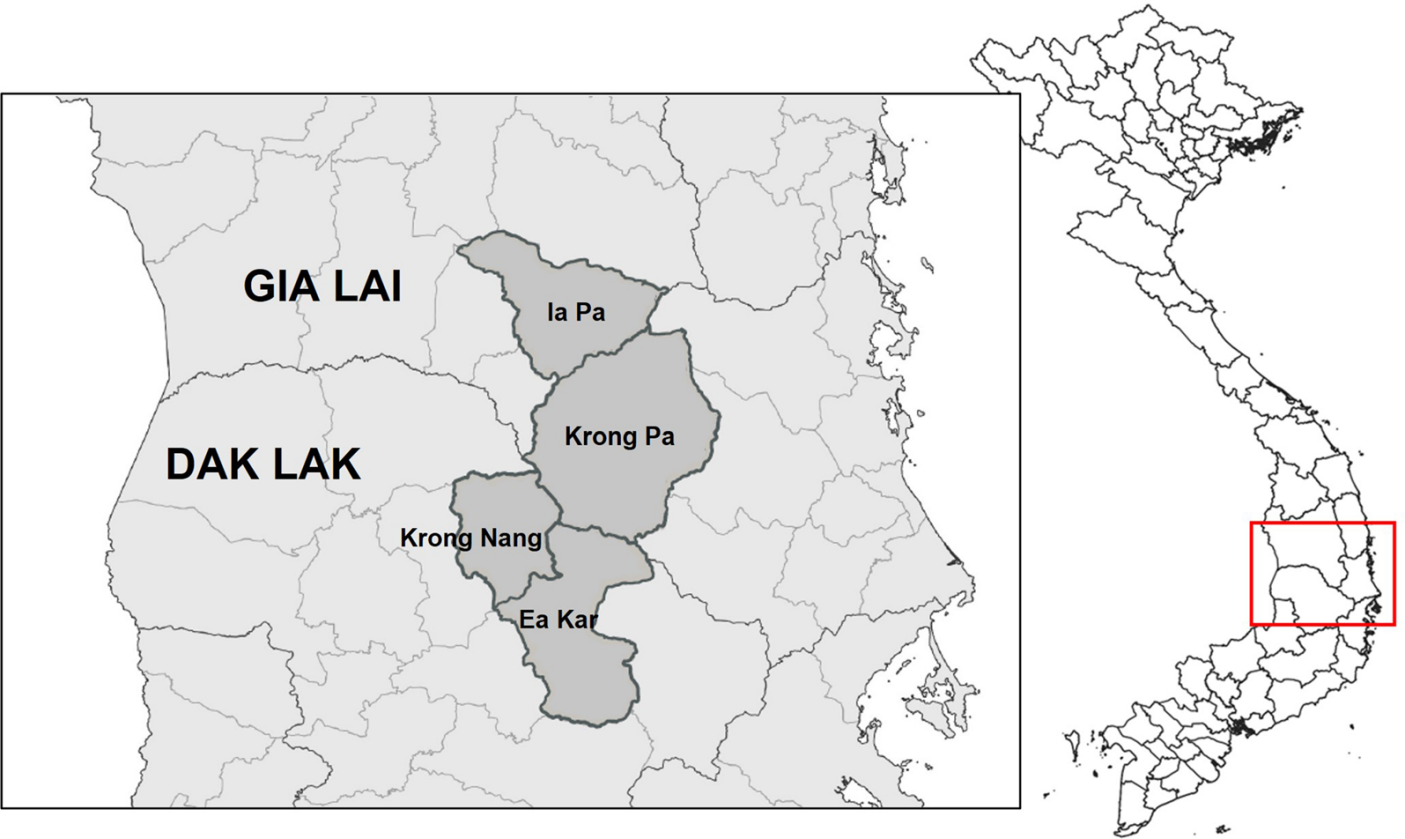
Study area. The study was conducted simultaneously in 33 CHC distributed across Ia Pa and Krong Pa districts (Gia Lai province) and Krong Nang and Ea Kar districts (Dak Lak). This map was developed for the purpose of this article

The study was conducted in four malaria endemic districts located along the border of Gia Lai (Krong Pa and Ia Pa districts) and Dak Lak (Ea Kar and Krong Nang districts) provinces, Central Highlands, Vietnam (Fig. 1). A total of 33 community health centres (CHC) were included: 21 in Gia Lai and 12 in Dak Lak. The area is characterized by a tropical climate and hilly geography with partially forested areas. The main occupation is agriculture in forest fields (maize, manioc, rice), which are often located far from villages of residence and require overnight stays in the forest. Malaria transmission mainly occurs in forested areas and is perennial with a peak between September and December and the lowest incidence from February to April. Both provinces have the highest burden of malaria in the country. In years 2018, 2019 and 2020, there were 946, 1804 and 530 malaria cases in Gia Lai and 733, 638 and 124 malaria cases in Dak Lak, respectively (National Institute of Malariology, Parasitology and Entomology, NIMPE). *Plasmodium falciparum* represented 70% of reported cases.

### Sample size

Due to the absence of conclusive preliminary evidence on false-negative RDT prevalence or *pfhrp2* deletions in the country, an initial sample size of 370 symptomatic *P. falciparum* patients per sampling domain was targeted as recommended in WHO guidelines [9]. This sample size would be adequate to demonstrate that a *pfhrp2/3* deletion prevalence is below the 5% threshold for an expected population prevalence of 3.2% with 95% confidence. The four districts across the two provinces were considered as a single sampling domain.

### Patients and enrolment procedures

Between June 2019 and June 2021 participants were recruited from individuals self-presenting at CHC with symptoms requiring a test for malaria, based on the criteria of the medical staff. Routine testing was conducted using SD Bioline Malaria Pf/Pv Antigen (Abbott) and LM, as further detailed below. Patients with confirmed *P. falciparum* diagnosis by LM and/or their parents/guardians were informed about the study objectives and invited to participate. Individuals with *P. vivax* malaria or other non-falciparum species by LM, including mixed infections, and those presenting signs or symptoms of severe malaria were excluded [12]. A clear explanation was provided in Vietnamese language and signed informed consent (or parental/guardian permission for those under 18 years of age and ≥ 12 months old and assent for those between 10 and 17 years of age) was obtained. For all consenting individuals, three dried blood spots (DBSs) containing 50 μl of blood each were obtained from a second finger prick, air-dried for 24 h and stored in silica gel containing bags, before being transferred to NIMPE (Hanoi, Vietnam) for molecular analysis. After sample collection, patients were treated for malaria as per Vietnam’s Ministry of Health (MoH) national guidelines, and asked to complete the short questionnaire on malaria symptoms and treatment, prevention habits and risk behaviour.

### Light microscopy

All microscopy evaluations at point-of-care were conducted by expert microscopists (WHO Level 1 or equivalent). Blood slides were stained with Giemsa and examined at a magnification of 1000×. Parasite density was determined by counting the number of asexual parasites per 200 white blood cells (WBCs) with a hand tally counter. If more than 500 parasites were counted before reaching 200 WBCs, the count was stopped after completion of the field. Density was expressed as the number of asexual parasites per µl of blood, calculated by dividing the number of asexual parasites by the number of WBCs and corrected by the estimated WBCs density (typically 8000 per µl). A blood slide was considered negative when examination of 1000 WBCs revealed no asexual parasites. All blood films were stored in boxes and shipped to NIMPE (Hanoi, Vietnam) for double reading by WHO Level 1 microscopists to confirm results from the field and adjust the final diagnosis, if necessary. A third reading was conducted only in case of discrepancy (i.e., disagreement in species identification, in positive vs negative, or in parasite density estimation > 25%).

### Quantitative PCR

DNA was extracted at NIMPE from 5 mm punches of DBS using QIAamp DNA 96 Blood kit (Qiagen), following manufacturer’s instructions and a final elution in 200 μl of water. A duplex quantitative real-time polymerase chain reaction (qPCR) targeting 18S ribosomal gene of *P. falciparum* and *P. vivax* was conducted to confirm species identified by LM [13]. Briefly, a 7.5 µl mix was prepared using primers and probes from QuantiNova Probe RT-PCR Kit (Qiagen) in a 7500 Real-Time PCR System (Applied Biosystems). Ct value > 40 was considered negative. Samples with no amplification in the Pf–Pv specific qPCR were checked for presence of other *Plasmodium* species using the generic QMAL qPCR assay [14].

### Genotyping of *pfhrp2* and *pfhrp3*

The regions covering exon 2 of both *pfhrp2* and *pfhrp3* genes were assessed by nested polymerase chain reaction (PCR) amplification using primers from Baker et al. [15] and HotStarTaq Plus Master Mix Kit (Qiagen), under the optimized cycling conditions described in Parr et al. [16]. DNA obtained from DBS of laboratory strains 3D7 (no deletions), Dd2 (*pfhrp2*-deletion; *pfhrp3*-wild type), HB3 (*pfhrp2*-wild type; *pfhrp3*-deletion) were used as positive controls (kindly provided by Pau Cisteró, ISGlobal, Barcelona). Nested PCR products were visualized in a 2% agarose gel and samples were considered positive if a band was observed in the 600-1000 bp range (in 3D7, expected products are 790 bp for *pfhrp2* and 698 bp for *pfhrp3*). DNA samples testing negative for *pfhrp2* and/or *pfhrp3* were further analysed by nested PCR of *P. falciparum* glutamine-rich protein (*pfglurp*) [17]. Negative result in *pfglurp* confirmatory PCR was interpreted as low quality/insufficient DNA and invalidated *pfhrp2* or *pfhrp3* negative result as evidence of deletion. A random selection of ≈ 10% of the samples (n = 40) was shipped for external quality assessment of *pfhrp2* and *pfglurp* nested PCRs at IMPE-Quy Nhon laboratory (Quy Nhon, Binh Dinh Province, Vietnam); results agreement was 100% (40/40) for both assays.

### *pfhrp2* sequencing

*pfhrp2-*exon2 PCR products were shipped to Genewiz (Suzhou, China) for DNA purification and bidirectional Sanger sequencing. Analysis was conducted in BioEdit 7.0.5. Quality of chromatograms of both forward and reverse sequences was inspected by eye, forward sequence and reverse complement of reverse sequence were aligned, and a consensus sequence was generated at overlapping regions. Nucleotide sequences were translated to amino acids and a multiple sequence alignment was performed using Clustal Omega in EMBL-EBI server (https://www.ebi.ac.uk/Tools/msa/clustalo/). *pfhrp2*sequence of the 3D7 strain (PF3D7_0831800) was included as reference.

The number and type of amino acid sequence repeats were identified based on the classification developed by Baker et al. [15]. The number of type 2 and type 7 repeats at each sequence was used to calculate type 2× type 7 score and classify sequences for their predicted sensitivity in RDT detection as type A (score > 100, “very sensitive”), type B (score 50–99; “sensitive”) or type C (< 50; “low or non-sensitive” group) [3, 15].

### HRP2 levels by ELISA

The levels of HRP2 protein were measured in all RDT negative/LM positive samples and a random selection of RDT positive samples. Protein elution was conducted from 2 × 5 mm punch of DBS (equivalent to 11.2 μl of blood or 5.6 μl of plasma, [18]), with a final elution in 1120 μl of buffer (1/200) [19]. Quantitative ELISA-based detection was conducted using Quantimal™ CELISA Ultra-sensitive PfHRP2 Malaria (Cellabs, Australia) according to manufacturer’s instructions [20]. Samples were tested in duplicate. Optical density (OD) values were read in a spectrophotometer at 450 nm/620 nm, and cut-off was set at negative control OD + 0.1. HRP2 concentration in plasma was estimated as previously described [21]. HRP2 concentration was calculated by interpolating OD in the standard curve of recombinant HRP2 provided in the kit (0.01–10 ng/ml). Samples with positive OD but below the limit of quantification of final standards were given a value of 0.01 ng/ml. Results were adjusted for specimen pre-dilution and expressed as estimated concentration in plasma.

### Definitions and statistical analysis

Prevalence of suspected false-negative HRP2-RDT results among symptomatic patients with *P. falciparum* malaria was determined from patient case report form data by dividing the number of patients testing RDT-negative and LM-positive by the total number of symptomatic *P. falciparum* cases. The prevalence of false-negative HRP2-RDT caused by *hrp2/3* deletions was determined as the number of *pfhrp2* or *pfhrp3* deletions divided by the total number of symptomatic *P. falciparum* cases. Differences between point estimates across sociodemographic and parasitological variables were tested using chi-square or Fisher’s exact test. Mann–Whitney/Kruskal Wallis test was used to compare parasite densities between population groups.

## Results

### Baseline characteristics of study participants

A total of 9456 patients were screened at CHCs during the study period (5366 [56.7%] in Dak Lak and 4090 [43.3%] in Gia Lai). Microscopy screening identified 370 patients with *P. falciparum* infections (3.9%) who were invited to participate. RDTs from SD Bioline Malaria Antigen Pf/Pv (Abbott) were positive for 353 patients, resulting in a suspected false-negative RDT prevalence among symptomatic cases of 4.6% (17/370). Molecular characterization of *Plasmodium* infection was assessed in 270 out of the 370 DBS samples collected. Of note, there was no selection bias based on RDT result, as samples were processed as they were collected and RDT results were not available to the laboratory until initial qPCR screening was completed. According to WHO guidelines for the surveillance of *hrp2/3* deletions, 270 cases should be sufficient to ensure the 95% confidence interval does not include the 5% *pfhrp2*-deletion warning threshold for an estimated *pfhrp2*-deletion population prevalence of 3.2% [9].

The baseline characteristics of 270 participants are provided in Table 1. Most individuals were adult males dedicated to farming and slash-and-burn agriculture as their main occupation. By district, Krong Pa accounted for 83.0% of the cases (224/270), followed by Krong Nang (24/270, 8.9%), Ea Kar (19/270, 7%) and Ia Pa (3/270, 1.1%). Headache and fever were the most common symptoms (264/265, 99.6%), followed by fatigue (245/265, 92.5%). Median parasite density was 6440 asexual parasites/μl (range 80–246,800; n = 264). Prevalence of false-negative RDT was 5.6% (15/270); 14 were from Krong Pa district and one from Ia Pa district.

**Table 1:** Baseline characteristics of participants (N = 270)

Variable	Gia Lai	Dak Lak	Overall
*P. falciparum* cases by LM	227	43	270
Number of CHC	15	12	27
Median age (range)	27 (7–57)	33 (21–58)	28 (7–58)
Sex			
Male	222 (97.8%)	43 (100%)	265 (98.1%)
Female	5 (2.2%)	0 (0)	5 (1.9%)
Occupation^a^			
Farmer	94 (42.0%)	20 (48.8%)	114 (43.0%)
Slash-and-burn cultivation	110 (49.1%)	13 (31.7%)	123 (46.4%)
Other	20 (8.9%)	8 (19.5%)	28 (10.6%)
Travel history in last 2 months			
Visited forest fields^a^	189 (84.4%)	41 (100%)	230 (86.8%)
Travelled outside village of residence^a^	25 (11.2%)	16 (39.0%)	41 (15.5%)
Use of bednets			
At home^a^	199 (88.8%)	37 (90.2%)	236 (89.1%)
In forest fields^a^	171 (76.3%)	28 (68.3%)	199 (75.1%)
Fever^a^	223 (99.6%)	41 (100%)	264 (99.6%)
RDT result			
*P. falciparum*	212 (93.4%)	43 (100%)	255 (94.4%)
*P. vivax* or mixed	0 (0)	0 (0)	0 (0)
False-negative for *P. falciparum*	15 (6.6%)	0 (0)	15 (5.6%)

^a^n = 265 (Gia Lai, n = 224; Dak Lak, n = 41)

### Molecular characterization of suspected false-negative RDT results

Duplex Pf-Pv qPCR was applied to all 270 samples. The screening confirmed 260 *P. falciparum* mono-infections, whereas 7 samples were *P. falciparum-P. vivax* co*-*infections (Table 2). Three samples were negative for both species and tested negative in the generic *Plasmodium* qPCR assay. Infections with false-negative RDT results were all *P. falciparum* mono-infections and individual details are provided in Additional file 1: Table S1. Parasite density by LM was lower in false-negative RDT result samples (2460 parasites/μl [interquartile range, IQR 1200–4800]; n = 14) as compared to RDT-positive cases (7160 parasites/μl [IQR 2280–20900]; n = 248; p = 0.005, Kruskal–Wallis). Similar results were found in qPCR, with higher Ct (i.e., lower parasite DNA concentration) in false-negative RDT cases as compared to RDT-positive cases (p = 0.028, Kruskal–Wallis; Table 2).

**Table 2:** Molecular characterization of *P. falciparum* cases, stratified by RDT result

	Pf positive RDT result	False-negative RDT result	All
DBS processed	255	15	270
Species qPCR			
*P. falciparum*	245 (96.1%)	15 (100%)	260 (96.3%)
*Pf–Pv* mixed infections	7 (2.7%)	0 (0)	7 (2.6%)
Negative	3 (1.2%)	0 (0)	3 (1.1%)
Ct (Pf), median [IQR]	28.1 [26.6–29.9]	30.0 [I27.5–31.4]	28.2 [26.7–30.0]
*hrp2/3* PCR positivity			
*pfhrp2 exon2*	252/252 (100%)	14/15 (93.3%)	266/267 (99.6%)
*pfhrp3 exon2*	252/252 (100%)	14/15 (93.3%)	266/267 (99.6%)
HRP2 plasma ng/mL, median [IQR]	54.5 [15.2–226.0]^a^	11.8 [2–14.9]	26.0 [11.9–209.7]

^a^n = 44

Fourteen out of the 15 suspected false-negative RDT results were successfully amplified at both *pfhrp2* and *pfhrp3* exon2 (Table 2 and Additional file 1: Table S1). The one sample which had no visible bands in both *pfhrp2* and *pfhrp3* was also negative when tested for *pfglurp* PCR amplification, suggestive of insufficient sensitivity of nested PCR methods (qPCR Ct of the sample = 39). Overall, the prevalence of false-negative HRP2-RDT caused by confirmed *hrp2/3* deletions among symptomatic *P. falciparum* cases was 0% (0/266).

HRP2 expression was assessed in all false-negative RDT and in a subset of 44 positive RDT samples. All samples had OD values above the negative control cut-off, but levels were significantly lower in the false-negative RDT group (p < 0.001; Kruskal–Wallis; Table 2 and Additional file 1: Fig. S1). There was a positive correlation between HRP2 levels and parasite density by LM (Pearson’s coefficient: 0.450, p < 0.001; Additional file 1: Fig. S2A) or qPCR Ct values (Pearson’s coefficient: − 0.657, p < 0.001; Additional file 1: Fig. S2B).

### HRP2 sequence variants

Genotyping of *pfhrp2* and *pfhrp3*-exon2 was also conducted for the remaining *P. falciparum* RDT-positive samples (n = 255). Bands were observed for both genes in all the remaining cases. Sequencing of exon2 was successful in 235/266 *pfhrp2* PCR products (Additional file 2: Data S1).

Three sequence variants were identified in the study population: VN1 (207/235, 88.1%), VN2 (24/235, 10.2%) and VN3 (4/235, 1.7%) (Table 3 and Additional file 1: Fig. S3). The type2 × type7 score and category is shown in Table 3. VN1 was classified as ‘C’ (predicted “low/no RDT sensitivity”), and VN2 and VN3 were classified as ‘B’ (predicted “sensitive”). The distribution of variants did not differ significantly by province (p = 0.465, Fisher’s exact). VN1 was found in all four study districts, VN2 in all except Ia Pa, and VN3 only in Krong Pa district. All false-negative RDT results with sequence data available (n = 13) corresponded to the predominant variant VN1 (Additional file 1: Table S1).

**Table 3:** Amino acid repeats in HRP2 sequence variants identified in Vietnam

Repeat id	Sequence	Sequence variant		
		VN1 (n = 207)	VN2 (n = 24)	VN3 (n = 4)
1	AHHAHHVAD	1	2	3
2	AHHAHHAAD	15	12	11
3	AHHAHHAAY	1^a^	2	1
4	AHH	0	0	1
5	AHHAHHASD	1	1	2
6	AHHATD	3	4	3
7	AHHAAD	2	6	5
8	AHHAAY	2	1	1
10	AHHAAAHHATD	1	2	2
12	AHHAAAHHEAATH	1	1	1
type2 × type7 score		30	72	55
type2 × type7 category		C	B	B

^a^Found in one sample only

## Discussion

This survey was used to determine the contribution of *hrp2/3* deletions to false-negative RDT results in Gia Lai and Dak Lak provinces between 2019 and 2021. Despite ≈ 5% of symptomatic cases confirmed by LM being negative by HRP2-RDT, there was no evidence of gene deletions in the surveyed population.

The rapid emergence of *hrp2/3*-deleted parasite populations in countries like Eritrea or Djibouti in recent years has raised alarms about the threat posed by parasite diagnostic escape to malaria control. In general, many malaria surveillance and/or clinical studies use HRP2-based RDT as a screening tool to identify malaria cases in human populations. This leads to an intrinsic selection bias of studied parasite population that has probably made difficult incidental and/or early detection of *hrp2*-deleted clones. In 2018, the WHO launched a reference protocol for the surveillance of *hrp2/3*-deletions to ensure elements were included in survey design [9]. All of the following were taken into account in the present survey: sampling in ≈ 10 health facilities across the sampling domain, quality-assured microscopy as a confirmatory test, confirmation of species and parasite density by qPCR, genotyping of both *pfhrp2* and *pfhrp3*, amplification of a confirmatory single-copy gene in the event of *hrp2/3* deletions and measurement of HRP2 concentration by serological analysis [6, 9]. In terms of sample size, molecular data was generated for 270 out of 370 participants initially recruited. The study was heavily affected in 2020 by both a marked reduction in malaria cases and the lockdowns caused by the SARS-CoV-2 pandemic in Vietnam, which made difficult the sample shipments and population movement to/from study sites. However, the final sample size had enough power for population prevalence of deletions up to 3.2%.

Molecular characterization of false-negative RDT results indicated that they were not caused by deletions in exon2 of *pfhrp2*. Although deletions can also occur around exon1 (exon1/2 region) [9], studies that genotyped this region show that almost all parasites lacking exon1/2 also lack exon2 [5, 22–24]. Moreover, previous molecular assays targeting exon1/2 can yield to spurious amplification of the paralogous gene [16]. The lower parasite densities found in false-negative RDT cases (further confirmed by lower HRP2 plasma levels) suggest that these infections could be below the test’s limit of detection (LOD). This hypothesis is not supported by LM parasitaemia—which was > 400 parasites/µl in all cases—but potential overestimation of parasitaemia by the LM-WBC count method cannot be excluded [25]. Interestingly, all false-negative RDT were caused by an HRP2 variant classified as category C—“low sensitivity”, according to predicted targeting of HRP2 epitope by RDT monoclonal antibodies [3, 15]. This variant was the most common overall and also present in most RDT positive samples, suggesting RDT escape properties are unlikely to explain negative test result. HRP2 sequence diversity was low with only three exon2 variants, probably due to focal transmission of a limited number of clones. Although this study targeted a large number of CHC within each district—to aim for maximum representativity of parasite population—this result highlights the risk of oversampling clonal populations when conducting *hrp2/3* surveys in very low transmission/pre-elimination areas using the same study design as for high transmission areas.

The survey has other limitations. First, it was not possible to assess the contribution of causes other than gene deletions to false-negative RDT, such as adherence to manufacturer’s instructions, transportation conditions, or storage conditions. User interpretation errors were ruled out, as CHC staff took pictures from all RDTs to be double checked by the study team. Second, the detection of parasites with *hrp2/3* gene deletions in polyclonal infections (i.e. infections with both *hrp2/3*-deleted and *hrp2/3*-wild type clones) may have been missed in the nested PCR approach.

In conclusion, there was no evidence of *P. falciparum* parasites carrying *pfhrp2* or *pfhrp3* gene deletions among symptomatic cases in Gia Lai and Dak Lak provinces, suggesting HRP2-RDTs remain effective for malaria case management. Given that parasites populations with *hrp2/3*-deletions have emerged rapidly in other countries [7], new surveys in these and other malaria-endemic provinces of Vietnam will be required in the future.

## Supplementary Information

**Additional file 1: Table S1.** Parasitological characteristics of samples with false-negative RDT result (n = 15). **Figure S1.** HRP2 levels in plasma, by RDT result. **Figure S2.** Correlation between HRP2 levels in plasma and parasite density. **Figure S3.** HRP2 sequence variants in *P. falciparum* infections from Gia Lai and Dak Lak provinces, Vietnam.

**Additional file 2: Data S1.** HRP2 exon 2 sequences.

## Data Availability

The authors confirm that the data supporting the findings of this study are available within the article and/or its supplementary materials. Any additional data that does not compromise the privacy of research participants is available from the corresponding author, ERV, upon reasonable request.

## Abbreviations

CHC: Community health centre
CI: Confidence interval
ELISA: Enzyme-linked immunosorbent assay
glurp: Glutamine-rich protein
HRP: Histidine-rich protein
IQR: Interquartile range
LOD: Limit of detection
NIMPE: National Institute of Malariology, Parasitology and Entomology
OD: Optical density
PCR: Polymerase chain reaction
qPCR: Quantitative real-time PCR
RDT: Rapid diagnostic test
WBC: White blood cells
WHO: World Health Organization

## References

[1] Howard RJ, Uni S, Aikawa M, Aley SB, Leech JH, Lew AM (1986). Secretion of a malarial histidine-rich protein (Pf HRP II) from *Plasmodium falciparum*-infected erythrocytes. J Cell Biol.

[2] UNITAID-World Health Organization. Malaria diagnostics landscape update. Geneva: World Health Organization; 2015. http://unitaid.org/assets/Malaria_Diagnostics_Landscape_Update_Fe_2015.pdf.

[3] Baker J, Ho MF, Pelecanos A, Gatton M, Chen N, Abdullah S (2010). Global sequence variation in the histidine-rich proteins 2 and 3 of *Plasmodium falciparum*: implications for the performance of malaria rapid diagnostic tests. Malar J.

[4] Gatton ML, Chaudhry A, Glenn J, Wilson S, Ah Y, Kong A (2020). Impact of *Plasmodium** falciparum* gene deletions on malaria rapid diagnostic test performance. Malar J.

[5] Gamboa D, Ho M-F, Bendezu J, Torres K, Chiodini PL, Barnwell JW (2010). A large proportion of *P. falciparum* isolates in the Amazon Region of Peru Lack pfhrp2 and pfhrp3: implications for malaria rapid diagnostic tests. PLoS ONE.

[6] Thomson R, Parr JB, Cheng Q, Chenet S, Perkins M, Cunningham J (2020). Prevalence of *Plasmodium falciparum* lacking histidine-rich proteins 2 and 3: a systematic review. Bull World Health Organ.

[7] Berhane A, Anderson K, Mihreteab S, Gresty K, Rogier E, Mohamed S (2018). Major threat to malaria control programs by *Plasmodium falciparum* lacking histidine-rich protein 2, Eritrea. Emerg Infect Dis.

[8] Iriart X, Menard S, Chauvin P, Mohamed HS, Charpentier E, Mohamed MA (2020). Misdiagnosis of imported falciparum malaria from African areas due to an increased prevalence of pfhrp2/pfhrp3 gene deletion: the Djibouti case. Emerg Microbes Infect.

[9] WHO. Protocol for estimating the prevalence of *pfhrp2/pfhrp3* gene deletions among symptomatic falciparum patients with false-negative RDT results. Geneva: World Health Organization; 2018. https://www.who.int/docs/default-source/malaria/mpac-documentation/mpac-oct2017-hrp2-deletion-protocol-session4.pdf?sfvrsn=2c9dfaf4_2.

[10] Goldlust SM, Thuan PD, Giang DDH, Thang ND, Thwaites GE, Farrar J (2018). The decline of malaria in Vietnam, 1991–2014. Malar J.

[11] Cunningham J. Update on *Plasmodium falciparum hrp2/3* gene deletions. Malaria Policy Advisory Committee Meeting, World Health Organization; 2017. https://www.who.int/malaria/mpac/mpac-mar2017-hrp2-3-deletions-session7-presentation.pdf.

[12] WHO (2012). Management of severe malaria: a practical handbook.

[13] Kamau E, Alemayehu S, Feghali KC, Saunders D, Ockenhouse CF (2013). Multiplex qPCR for detection and absolute quantification of malaria. PLoS ONE.

[14] Wampfler R, Mwingira F, Javati S, Robinson L, Betuela I, Siba P (2013). Strategies for detection of *Plasmodium* species gametocytes. PLoS ONE.

[15] Baker J, McCarthy J, Gatton M, Kyle DE, Belizario V, Luchavez J (2005). Genetic diversity of *Plasmodium falciparum* histidine-rich protein 2 (PfHRP2) and its effect on the performance of PfHRP2-based rapid diagnostic tests. J Infect Dis.

[16] Parr JB, Anderson O, Juliano JJ, Meshnick SR (2018). Streamlined, PCR-based testing for pfhrp2- and pfhrp3-negative *Plasmodium falciparum*. Malar J.

[17] Snounou G (2002). Genotyping of *Plasmodium* spp. nested PCR. Methods Mol Med.

[18] Corran PH, Cook J, Lynch C, Leendertse H, Manjurano A, Griffin J (2008). Dried blood spots as a source of anti-malarial antibodies for epidemiological studies. Malar J.

[19] San NN, Kien NX, Manh ND, Van Thanh N, Chavchich M, Binh NTH (2022). Cross-sectional study of asymptomatic malaria and seroepidemiological surveillance of seven districts in Gia Lai province, Vietnam. Malar J.

[20] Mihreteab S, Anderson K, Pasay C, Smith D, Gatton ML, Cunningham J (2021). Epidemiology of mutant *Plasmodium falciparum* parasites lacking histidine-rich protein 2/3 genes in Eritrea 2 years after switching from HRP2-based RDTs. Sci Rep.

[21] WHO. Methods manual for laboratory quality control testing of malaria RDTs. Geneva: World Health Organization; 2020. https://www.who.int/publications/m/item/methods-manual-for-laboratory-quality-control-testing-of-malaria-rdts.

[22] Bharti PK, Chandel HS, Ahmad A, Krishna S, Udhayakumar V, Singh N (2016). Prevalence of pfhrp2 and/or pfhrp3 gene deletion in *Plasmodium falciparum* population in eight highly endemic States in India. PLoS ONE.

[23] Gupta H, Matambisso G, Galatas B, Cisteró P, Nhamussua L, Simone W (2017). Molecular surveillance of pfhrp2 and pfhrp3 deletions in *Plasmodium falciparum* isolates from Mozambique. Malar J.

[24] Kumar N, Pande V, Bhatt RM, Shah NK, Mishra N, Srivastava B (2013). Genetic deletion of HRP2 and HRP3 in Indian *Plasmodium falciparum* population and false negative malaria rapid diagnostic test. Acta Trop.

[25] Alves ER, Gomes LT, Ribatski-Silva D, Mendes CRJ, Leal-Santos FA, Simões LR (2014). Assumed white blood cell count of 8,000 cells/μL overestimates malaria parasite density in the Brazilian Amazon. PLoS ONE.

