# Supplementary material for: Surveillance of pfhrp2 and pfhrp3 gene deletions among symptomatic Plasmodium falciparum malaria patients in Central Vietnam
Source: Malar J. 2022 Dec 5;21:371. doi: 10.1186/s12936-022-04399-w (PMC9724378; doi:10.1186/s12936-022-04399-w)

**Surveillance of *pfhrp2* and *pfhrp3* gene deletions among symptomatic *Plasmodium falciparum* malaria patients in Central Vietnam**

**Additional file 1**

Table S1. Parasitological characteristics of samples with false-negative RDT result (n=15).

Figure S1. HRP2 levels in plasma, by RDT result.

Figure S2. Correlation between HRP2 levels in plasma and parasite density.

Figure S3. HRP2 sequence variants in P. falciparum infections from Gia Lai and Dak Lak provinces, Vietnam.

Data S1. HRP2 exon 2 sequences (.fasta).

**Table S1. Parasitological characteristics of samples with false-negative RDT result (n=15).**

| ID | Age (years) | District | Microscopy | | qPCR 18S | | *hrp2*/HRP2 | | | | *hrp3* | *glurp* |
| --- | --- | --- | --- | --- | --- | --- | --- | --- | --- | --- | --- | --- |
|  |  |  | Species | parasites/μl | Species | Ct value | PCR | sequence variant | type2x7 score/category | plasma conc (ng/mL) | PCR | PCR |
| I032 | 25 | Ia Pa | Pf | 1880 | Pf | 31.0 | positive | VN1 | 30 / D | 6.0 | positive | . |
| K077 | 12 | Krong Pa | Pf | 1280 | Pf | 32.4 | positive | VN1 | 30 / D | 11.9 | positive | . |
| K101 | 37 | Krong Pa | Pf | 800 | Pf | 31.4 | positive | VN1 | 30 / D | 2* | positive | . |
| K115 | 31 | Krong Pa | Pf | 1200 | Pf | 27.5 | positive | VN1 | 30 / D | 11.8 | positive | . |
| K123 | 21 | Krong Pa | Pf | 3000 | Pf | 28.5 | positive | VN1 | 30 / D | 19.7 | positive | . |
| K137 | 26 | Krong Pa | Pf | 4800 | Pf | 28.5 | positive | VN1 | 30 / D | 12.7 | positive | . |
| K138 | 10 | Krong Pa | Pf | 2800 | Pf | 39.3 | negative | . | . | 2* | negative | negative |
| K143 | 21 | Krong Pa | Pf | 6320 | Pf | 30.9 | positive | VN1 | 30 / D | 2* | positive | . |
| K162 | 20 | Krong Pa | Pf | 6480 | Pf | 27.3 | positive | VN1 | 30 / D | 19.9 | positive | . |
| K238 | 36 | Krong Pa | Pf | . | Pf | 30.0 | positive | . | . | 2* | positive | . |
| K241 | 28 | Krong Pa | Pf | 2920 | Pf | 26.9 | positive | VN1 | 30 / D | 9.3 | positive | . |
| K253 | 32 | Krong Pa | Pf | 2120 | Pf | 27.7 | positive | VN1 | 30 / D | 14.9 | positive | . |
| K259 | 26 | Krong Pa | Pf | 320 | Pf | 30.8 | positive | VN1 | 30 / D | 11.8 | positive | . |
| K302 | 22 | Krong Pa | Pf | 400 | Pf | 31.6 | positive | VN1 | 30 / D | 2* | positive | . |
| K303 | 46 | Krong Pa | Pf | 24160 | Pf | 26.9 | positive | VN1 | 30 / D | 275.3 | positive | . |

* *positive below the limit of quantification.*

**Figure S1. HRP2 levels in plasma, by RDT result.** Median and interquartile range are indicated with horizontal lines. Distributions were compared using Kruskal-Wallis test.

**
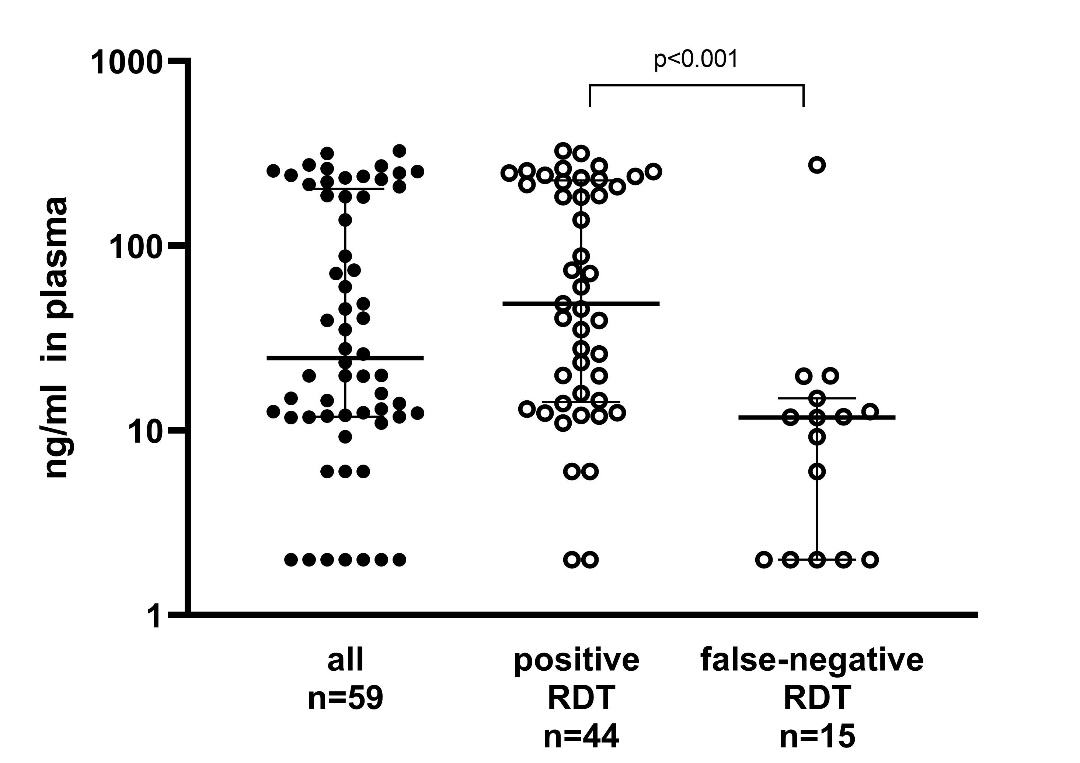
**

**Figure S2. Correlation between HRP2 levels in plasma and parasite density.** A) microscopy, B) 18S rRNA qPCR.

**
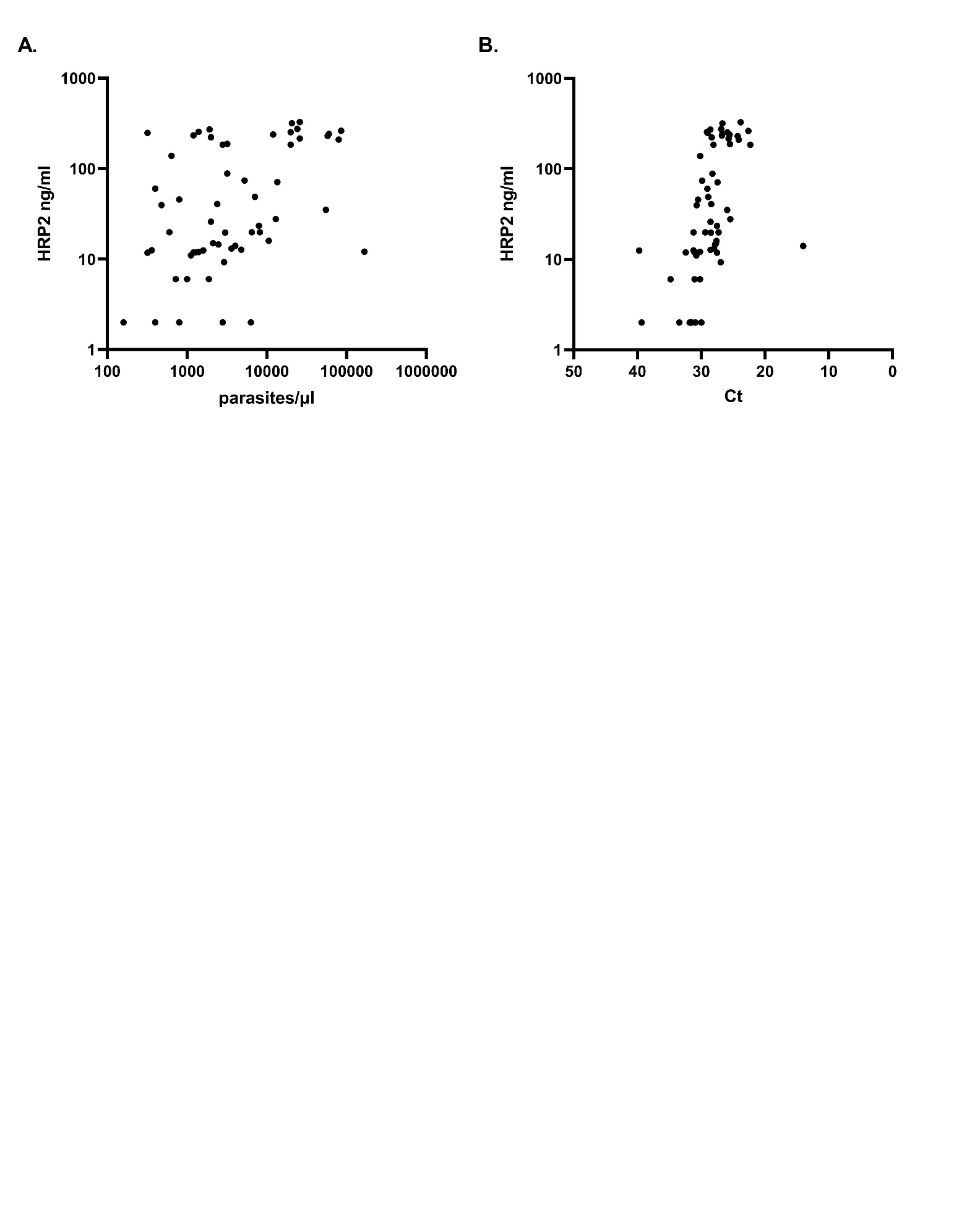
**

**Figure S3. HRP2 sequence variants in *P. falciparum* infections from Gia Lai and Dak Lak provinces, Vietnam.** The diagram shows the location and type of aminoacid repeats for each sequence.


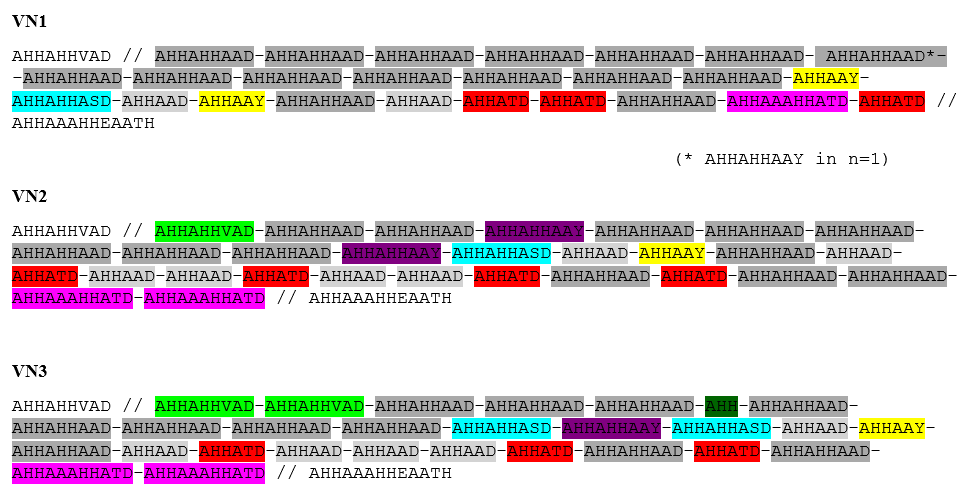

Supplement: Supplementary file 1 — Additional file 1: Table S1. Parasitological characteristics of samples with false-negative RDT result (n = 15). Figure S1. HRP2 levels in plasma, by RDT result. Figure S2. Correlation between HRP2 levels in plasma and parasite density. Figure S3. HRP2 sequence variants in P. falciparum infections from Gia Lai and Dak Lak provinces, Vietnam. [file 12936_2022_4399_MOESM1_ESM.docx]
